# Antimicrobial efficacy and cytotoxic assessment of plasma-activated water generated by a dielectric barrier discharge microbubble system

**DOI:** 10.3389/fmicb.2026.1746535

**Published:** 2026-04-01

**Authors:** Raquel Oliveira Ferreira, Danielle C. F. S. Spigarollo, Iolanda Cristina Silveira Duarte, Elaine C. de Oliveira, Nilson Cristino da Cruz

**Affiliations:** 1 Laboratory of Technological Plasma, Institute of Science and Technology, São Paulo State University (UNESP), Sorocaba, São Paulo, Brazil; 2 Laboratory of Applied Microbiology, Federal University of São Carlos (UFSCar), Sorocaba, São Paulo, Brazil; 3 Laboratory of Neuroscience, University of Campinas (Unicamp), Campinas, São Paulo, Brazil

**Keywords:** antimicrobial activity, cold atmospheric plasma, cytotoxicity, DBD plasma, microbubble diffusion, physicochemical characterization, plasma-activated water, reactive species

## Abstract

The growing threat of antimicrobial resistance has intensified the search for alternative disinfection strategies. Plasma-activated water (PAW), enriched with reactive oxygen and nitrogen species (RONS), has emerged as a promising non-antibiotic antimicrobial approach. In this study, PAW was generated using a bench-scale dielectric barrier discharge (DBD) reactor coupled with a microbubble diffusion system, operating with compressed ambient air at 8–9 kV, frequencies of 40 kHz, 1.25 W, air flow rate of 5 L/min with activation times of 7 and 14 min. In the generated PAW, ROS were produced in higher abundance than RNS, with hydrogen peroxide (H_2_O_2_) and ozone (O_3_) representing the dominant species contributing to the antimicrobial activity, alongside detectable levels of nitrite (NO_2_^–^), nitrate (NO_3_^–^), and nitrous acid (HNO_2_). Physicochemical characterization included measurements of pH, oxidation-reduction potential (ORP), conductivity, and total dissolved solids (TDS), as well as qualitative analysis of RONS using UV–Vis spectrophotometry (190–400 nm), for detection of diagnostic wavelengths, with quantitative assessment based on colorimetric methods for H_2_O_2_, NO_2_^–^, and NO_3_^–^, and a photometric assay for O_3_. Antimicrobial activity was evaluated against *Staphylococcus aureus* (ATCC 6538), *Escherichia coli* (ATCC 25922), and *Candida albicans* (ATCC 10231) using PAW generated at 7 and 14 min of activation. In addition, cytotoxicity was evaluated in L929 fibroblasts following ISO 10993-5 criteria, and cell viability remained above the 70% threshold after exposure to PAW. Morphological assessments were performed in both L929 and B16F10 cells using fluorescence microscopy to examine potential differential cellular responses. These findings indicate that longer activation times enhanced antimicrobial activity while preserving fibroblast viability, supporting the potential of PAW as an effective and safe alternative for biomedical applications.

## 1 Introduction

The global dissemination of antimicrobial-resistant microorganisms has become a major public health threat, with bacterial antimicrobial resistance (AMR) directly causing 1.27 million deaths and contributing to 4.95 million deaths globally in 2019, with mortality rates projected to increase by nearly 70% by 2050 compared to 2022 levels (CDC, 2019; Murray et al., 2022; Naghavi et al., 2024). This phenomenon threatens to compromise the progress of modern medicine, including procedures such as surgeries, chemotherapy, and immunosuppressive therapies, which rely heavily on the effectiveness of antimicrobial agents.

This crisis is primarily driven by the excessive and inappropriate use of antimicrobial agents. The misuse of antibiotics creates selective pressure favoring the emergence of multidrug-resistant bacterial strains, while inappropriate antifungal use similarly drives the development of antifungal-resistant fungal pathogens, with invasive fungal infections accounting for approximately 150 million cases and 3.8 million deaths annually (Liu et al., 2025; Mudenda and Mudenda, 2024). Several alternative strategies have been investigated, including bacteriophage-derived enzymes (endolysins), antimicrobial peptides, bacteriocins, and natural compounds such as plant-derived polyphenols (Ghosh et al., 2019; Mahlapuu et al., 2016). Although promising, these approaches still face significant limitations including cytotoxicity, challenges in large-scale production, high manufacturing costs, and lack of regulatory approval for clinical use (Taati Moghadam et al., 2025; Zheng et al., 2025), while conventional antibiotics continue to be widely used due to their availability, proven efficacy, and relatively low cost. However, a sharp decline has been observed in the development of new antibiotic classes, along with a shortening of the interval between the introduction of new drugs and the emergence of resistance. Different pathogen groups employ distinct resistance mechanisms: Gram-negative bacteria (e.g., *Escherichia coli*) rely primarily on their double membrane structure as a permeability barrier, alongside expression of efflux pumps and production of β-lactamases that hydrolyze antibiotics before reaching their targets; Gram-positive bacteria (e.g., *Staphylococcus aureus*) develop resistance through target site modifications (such as altered penicillin-binding proteins in methicillin-resistant *S. aureus* (MRSA), biofilm formation that restricts drug penetration, and acquisition of resistance genes via horizontal gene transfer; and fungi (e.g., *Candida albicans*) exhibit resistance through upregulation of efflux pumps, alterations in drug target enzymes (such as Erg11 in azole resistance), and biofilm formation, leading to invasive infections with high mortality rates, particularly in immunocompromised patients (Han et al., 2022; Pang and Kan, 2024; Sharma et al., 2024; Shen et al., 2016; Zheng et al., 2025). Given this scenario, there is an urgent need to develop new antimicrobial agents that are effective, safe, and act through mechanisms distinct from those employed by conventional agents. Among the emerging alternatives, plasma-activated water (PAW) has gained increasing attention as a non-antibiotic strategy with broad-spectrum antimicrobial potential (Droste et al., 2024; Han et al., 2022; Milhan et al., 2022; Tonmitr and Yonesu, 2023).

Technologies based on non-thermal atmospheric pressure plasma (NTAPP) have drawn growing interest due to their versatility and effectiveness in biomedical applications (Liao et al., 2017). This interest is largely attributed to its unique ability to generate a broad range of ionized and neutral reactive species under ambient conditions (Angelina Cullen et al., 2025; Ghorui et al., 2024; Kim et al., 2014; Tian et al., 2015). Among these, reactive oxygen and nitrogen species (RONS) stand out for their potent oxidative effects on biological systems. Unlike conventional disinfectants such as sodium hypochlorite (>500 ppm) and hydrogen peroxide (3–35%), plasma generates RONS in situ at micromolar concentrations with short lifetimes (microseconds to milliseconds), allowing localized antimicrobial action without high chemical loads or toxic residues (Droste et al., 2024; Miranda et al., 2023), thereby enabling diverse applications in both sterilization and therapeutics (Oh et al., 2016a; Szili et al., 2015). One of the most relevant advances stemming from NTAPP is the development of PAW. Distinguished by its unique antimicrobial and bioactive properties, PAW offers functional advantages over conventional chemical disinfectants or pure water, enabling its application in medical treatments (Semmler et al., 2020; Xu et al., 2023), dentistry (Milhan et al., 2022), agriculture (Kooshki et al., 2023; Tonmitr and Yonesu, 2023), and food safety (Patra et al., 2022; Sawangrat et al., 2022). Generated through the interaction between cold plasma and water, PAW becomes enriched with a complex array of RONS, including hydrogen peroxide (H_2_O_2_), and ozone (O_3_) (Liu et al., 2019; Liu et al., 2020; Oh et al., 2016b; Xu et al., 2023), as well as short-lived species such as nitrite (NO_2_^–^), nitrate (NO_3_^–^), superoxide (O_2_), nitric oxide (NO•) and hydroxyl radicals (•OH) (Lin et al., 2019; Oh et al., 2016b; Wu et al., 2017). These species are responsible for a wide range of biological effects, extending their applicability beyond microbial inactivation to more advanced domains such as seed germination and contributing to innovative therapeutic approaches, making PAW a potential candidate for clinical applications such as wound disinfection and surface sterilization, although regulatory approval, production scale-up, and *in vivo* safety validation remain as key challenges for widespread clinical adoption (Chiappim et al., 2021; Deng et al., 2025; Lee et al., 2022; Pavlovich et al., 2013). The antimicrobial action of RONS involves oxidative and nitrosative damage to bacterial cells. Species such as H_2_O_2_, O_3_, •OH, and O_2_^–^ induce lipid peroxidation and membrane disruption, while NO_2_^–^, NO_3_^–^, and peroxynitrite (ONOO^–^) cause protein oxidation and DNA damage, ultimately leading to cell death through multi-target mechanisms (Nikolaou et al., 2025).

PAW can be generated using various plasma sources, such as pin-to-water systems, gliding arc plasma jets (GAPJ), and especially dielectric barrier discharge (DBD), known for its stability and efficient transfer of reactive species to liquids (Azevedo Neto et al., 2024; Miranda et al., 2024; Punith et al., 2024; Xu et al., 2023). Systems like KiPen, a portable cold plasma device, have also been applied in dental and medical contexts for liquid activation (Winter et al., 2015). However, many setups require pure gases like argon or helium, which increase costs (∼$14/m^3^ for helium) and limit scalability (Mohades et al., 2020). To overcome these challenges, bench-scale DBD systems using compressed ambient air have emerged as low-cost (no gas procurement), practical, and energy-efficient (2–4x lower energy consumption) alternative (Roy et al., 2023). These systems show promise for clinical integration, enabling on-demand PAW production for use in procedures such as disinfection, wound care, and therapeutic applications.

In parallel with the development of such systems, there is a growing interest in understanding the determinants of RONS production, particularly in the context of plasma-PAW. However, significant gaps remain regarding air-DBD systems with bubbling configurations. While plasma jets and gliding arc systems have been extensively characterized, air-DBD coupled with bubbling technology remains underexplored, despite advantages in cost-effectiveness, scalability, and operational control. Several recent studies have contributed to advancing the understanding of PAW by exploring specific aspects of its generation and chemical composition, while some have specifically focused on its potential to enhance antimicrobial effects. Zhang et al. (2023) investigated the formation of reactive species using different types of plasma jets, carrier gases, and exposure times, highlighting the chemical complexity of plasma-liquid interactions. Tonmitr and Yonesu (2023) combined different discharge frequencies with microwave plasma to enhance the efficiency of RONS generation. Similarly, Xu et al. (2023) evaluated the physicochemical properties and anticancer effects of air DBD in solutions treated with ozone-enriched gas. The results showed that both gaseous and aqueous reactive oxygen (ROS) and nitrogen species (RNS) vary according to discharge modes and plasma configurations, leading to significant effects on the viability of cancer cells. Azevedo Neto et al. (2024) conducted a comprehensive characterization of PAW produced by a hybrid system combining DBD and GAPJ, systematically emphasizing the role of activation parameters in RONS formation. Miranda et al. (2024) evaluated the influence of different carrier gases on PAW composition and antimicrobial performance, highlighting the critical importance of plasma configuration and gas-liquid interaction dynamics and compressed air being more effective than argon and helium for antimicrobial properties. Droste et al. (2024) demonstrated that the antimicrobial effect of PAW and plasma-activated saline is strongly related to the oxidative potential generated during activation, significantly affecting Gram-positive and Gram-negative bacterial suspensions. Sampaio et al. (2022) also contributed by showing how pH influences both the antibacterial potential and cytotoxicity of plasma-activated liquids produced by different systems. Their results revealed significant antimicrobial activity against *E. coli*, with safe application achieved under specific physicochemical conditions using a gliding arc system. Additionally, Chiappim et al. (2021) using tap water treated with a plasma jet in a forward vortex flow reactor showed significant reductions in viable cells, confirming antimicrobial activity against *S*. *aureus* and *E. coli.*

These studies have significantly advanced the field by characterizing various PAW generation systems and their antimicrobial properties. However, comprehensive investigations integrating DBD-based PAW generation using compressed air with bubbling technology, systematic physicochemical characterization, broad-spectrum antimicrobial evaluation (including fungi), and cytotoxicity analysis remain limited. Most existing studies focus on plasma jets, gliding arc systems, or DBD with expensive carrier gases, while air-DBD coupled with bubbling configurations remains underexplored despite its practical advantages. The bubbling configuration enhances gas-liquid interaction, enabling greater control over operational parameters and improved reproducibility. Understanding the relationships between plasma parameters (discharge frequency, activation time), chemical composition, and biological effects is crucial, as RONS, while essential for antimicrobial action, may also exhibit cytotoxic effects on eukaryotic cells. This understanding is fundamental to optimizing treatment conditions and enhancing PAW efficacy against microbial pathogens for healthcare applications.

This experimental study is outlined as follows. First, the experimental setup is presented, based on a bench-scale DBD system coupled with bubbling technology. To establish optimal discharge conditions, treatment time was investigated by analyzing physicochemical parameters including oxidation-reduction potential (ORP), conductivity (κ), and total dissolved solids (TDS). The RONS were qualitatively assessed using ultraviolet-visible (UV-Vis) spectroscopy. Based on these physicochemical analyses, two activation times (7 and 14 min) were selected for subsequent biological assays: 7 min as an intermediate condition and 14 min representing enhanced RONS generation. The antimicrobial activity of PAW was evaluated against representative pathogens: *Staphylococcus aureus* (ATCC 6538) as a Gram-positive model, *Escherichia coli* (ATCC 25922) as a Gram-negative model, and *Candida albicans* (ATCC 10231) as a fungal model, considering microbial growth over different activation and exposure times. Acute cytotoxicity (24-h exposure) was assessed using L929 fibroblasts following ISO 10993–5 biocompatibility standards. Additionally, fluorescence microscopy was employed to examine potential differential cellular responses in B16F10 melanoma cells to investigate biocompatibility for biomedical applications. This study establishes systematic correlations between operational parameters, physicochemical properties, and biological outcomes for air-DBD bubbling systems, addressing existing gaps and advancing their potential for clinical applications.

## 2 Materials and methods

### 2.1 Plasma-activated water generation

#### 2.1.1 Reactor setup and activation process

**FIGURE 1:**
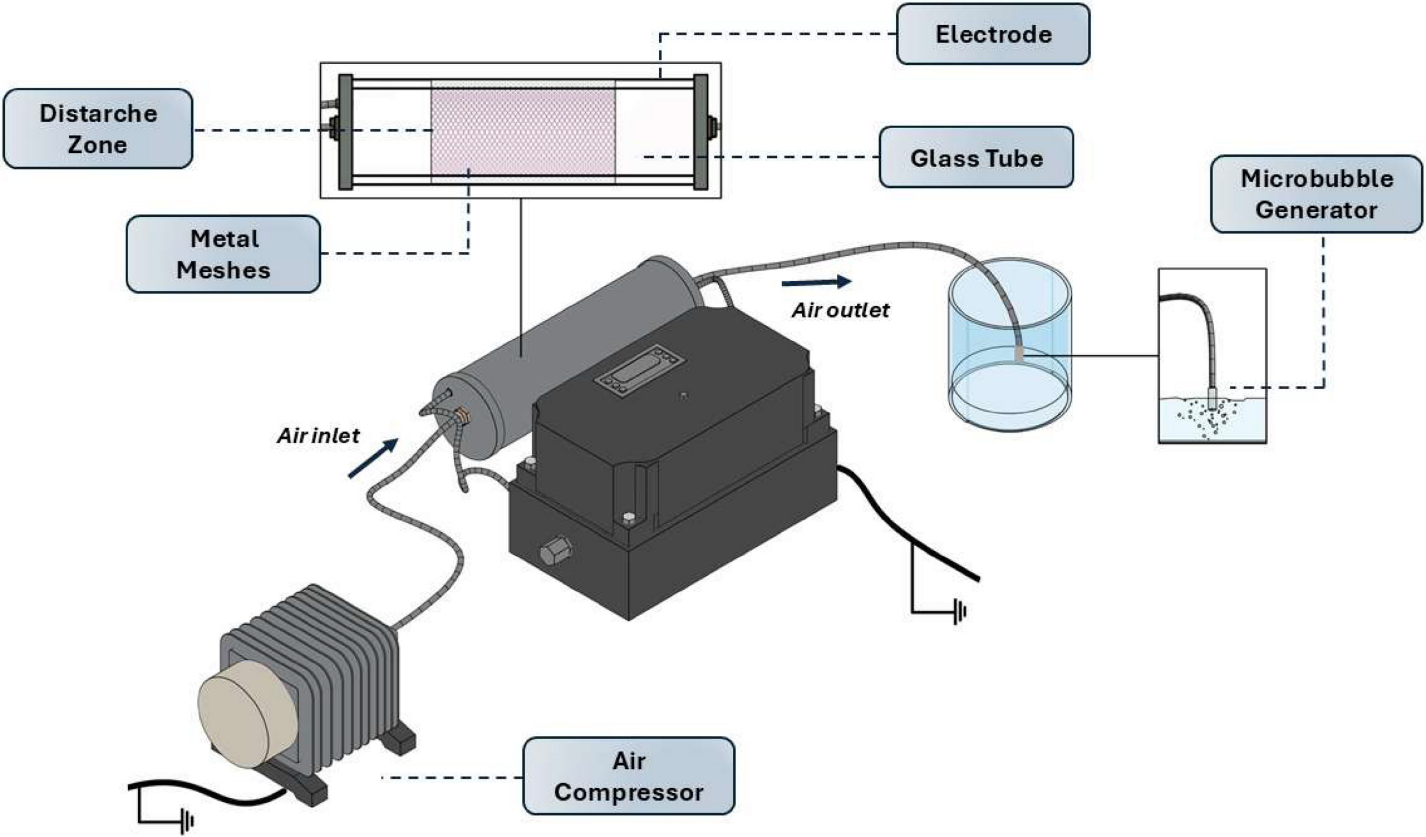
Schematic of the DBD plasma setup used for the generation of plasma-activated water.

The PAW was generated using a bench-scale DBD reactor coupled with a microbubble diffusion system, designed to enhance gas–liquid interaction during plasma exposure. The system consisted of a glass tube containing two parallel stainless-steel mesh electrodes, with one covered by a dielectric barrier, connected to a high-voltage AC power supply (Figure 1). The discharge zone was established between the electrodes, generating non-thermal plasma at atmospheric pressure. The reactor operated at approximately 8–9 kV peak-to-peak voltage, 40 kHz frequency, with 1.25 W power dissipation.

Compressed ambient air was used as the working gas and supplied by an external air compressor at a flow rate of 5 L/min. Before reaching the liquid phase, the air passed through the discharge chamber and was subsequently directed into the water via a stainless-steel microbubble diffuser. The bubbling configuration significantly increases the gas-liquid interfacial area, enhancing mass transfer of plasma-generated reactive species into water and promoting solution homogeneity.

Samples of 50 mL of deionized water were activated using a DBD system coupled with a bubbling technology consisting of a metallic component designed to reduce bubble size, thereby enhancing the transfer efficiency of the gas and the reactive species generated by the plasma into the water. The initial physicochemical properties of the deionized water used were: pH = 7.34 ± 0.03, ORP = 32.5 ± 0.2 mV, conductivity = 1.09 ± 1% μS⋅cm^−1^, and TDS = 0.429 ± 0.050 mg⋅L^−1^. All experiments were conducted at room temperature (approximately 25°C).

#### 2.1.2 Physicochemical and chemical characterization

These measurements were performed using a Metrohm 913 multi-parameter meter at 25 °C. Reactive species in PAW were identified using UV-Vis absorption the measurements of which were performed using a BEL Engineering UV-M51 single-beam spectrophotometer (BEL, Italy), equipped with quartz cuvettes (1 cm path length), complemented by commercial colorimetric test strips (Bartovation, United States) for quantification of NO_2_^–^ (detection range: 0.5–80 mg/L), NO_3_^–^ (10–500 mg/L), and H_2_O_2_ (0.5–25 mg/L). Test strips were immersed in PAW samples immediately after activation, and concentrations were determined by comparing color changes against manufacturer-provided calibration charts. Ozone (O_3_) concentration was measured using the Exact Micro 20 multi-parameter photometer (Industrial Test Systems, United States) based on the indigo trisulfonate method (Standard Method 4500-O_3_ B), with detection range of 0.01–0.25 mg/L. Furthermore, the concentration of nitrous acid (HNO_2_) was estimated based on the NO_2_^–^ concentration and the pH values of the solution, using the following equation (Sampaio et al., 2022):


$$\left[H\,N\,O_{2}\right]=\frac{{\left[N\,O_{2}^{-}\right]}_{t\,o\,t\,a\,l}}{1+10^{\left(p\,H-p\,K_{a}\right)}}$$

where: [NO_2_^–^] is the NO_2_^–^ concentration, pH is the pH of the PAW, and pKa is the acidity constant of HNO_2._

### 2.2 Assessment of antimicrobial activity of PAW

Reference strains of the Gram-positive bacterium *Staphylococcus aureus* (ATCC 6538), Gram-negative bacterium *Escherichia coli* (ATCC 25922), and the yeast *Candida albicans* (ATCC 10231) were included in this study were obtained from Plastlabor (Rio de Janeiro, RJ, Brazil) and included in this study. Bacterial strains were cultured on tryptic soy agar (TSA), and the yeast was cultured on Sabouraud dextrose agar (SDA). The plates were incubated aerobically at 35 °C for 24 h. Standardized suspensions (OD_600_ = 1.0) of each microorganism were prepared in sterile saline solution (0.9% NaCl) using a spectrophotometer (HACH DR 5000), set to a wavelength (λ) of 600 nm and an optical density (OD) of 1.0, The approximate concentrations were 1.5 × 10^9^ CFU/mL for *S. aureus*, 8.0 × 10^8^ CFU/mL for *E. coli*, and 1.0 × 10^5^ CFU/mL for *C. albicans*. The antimicrobial activity of PAW was evaluated using two experimental groups: (a) PAW (sterilized immediately after activation), and (b) sterilized deionized water (non-activated), used as a negative control. Microbiological assays were conducted immediately following PAW activation. The microbial viability was assessed by monitoring microbial growth, 1.0 mL of PAW (activated for 7 or 14 min) was added to 1.0 mL of the standardized microbial suspension in sterile test tubes. The mixtures were maintained in contact for the corresponding activation time (7 or 14 min). Matched activation and exposure times were employed to assess the combined effect of RONS concentration and contact duration on antimicrobial efficacy, consistent with previous PAW studies (Fernández-Gómez et al., 2023; Miranda et al., 2023). Subsequently the exposure period, 8.0 mL of Mueller-Hinton Broth (MHB) was added to each sample. The tubes were gently homogenized and incubated at 35 °C for 24, 48, and 72 h. Microbial growth was monitored using spectrophotometric readings at OD_600_. The percentage of microbial reduction was calculated using the following equation:


MicrobialReduction(%)=

$$\left(\frac{C\,F\,U/m\,L\,c\,o\,n\,t\,r\,o\,l-C\,F\,U/m\,l\,t\,r\,e\,a\,t\,e\,d}{C\,F\,U/m\,l_{c\,o\,n\,t\,r\,o\,l}}\right)\times 100$$

All antimicrobial experiments were performed with three technical replicates (*n* = 3) for each experimental condition. Data was analyzed using one-way, two-way, and three-way ANOVA to evaluate the effects of treatment, activation time, and incubation period, including their interactions. For pairwise comparisons, Welch’s *t*-test was applied to account for unequal variances between groups. A significance level of *p* < 0.05 was adopted for all analyses. Energy efficiency of the antimicrobial process was evaluated by calculating the log reduction per kilowatt-hour (log/kWh) of dissipated energy. Log reduction values were derived from percentage inhibition data at 24 h using the formula: log reduction = −log_10_(1 − inhibition/100). Energy consumption was calculated as: Energy (kWh) = Power (kW) × Time (hours), where power was 0.00125 kW (1.25 W) at 40 kHz. Energy efficiency was determined as: Efficiency (log/kWh) = log reduction / energy consumption.

### 2.3 Cytotoxicity and morphological analysis

The cytotoxicity of PAW was assessed following ISO 10993–5:2009 guidelines. The cell lines used were B16F10 (murine melanoma) and L929 (murine fibroblast). After reaching 80% confluence, adherent cells were detached using trypsin-EDTA solution (Cultilab, Brazil), and the concentration was adjusted to 1 × 10^4^ cells/mL. Cells were seeded in 96-well plates for viability assays and in 24-well plates for morphological analysis and incubated at 37 °C with 5% CO_2_ for 24 h to allow complete adhesion.

On the following day, 50 mL of distilled water were plasma-activated for 7 and 14 min, then sterile-filtered (0.22 μm). PAW was mixed with complete Dulbecco’s Modified Eagle Medium DMEM (1:1, v/v). The culture medium was removed from the wells, and cells were exposed to the PAW-containing medium for 30 min. After exposure, the PAW medium was removed and replaced with fresh complete DMEM. The plates were incubated for an additional 24 h prior to the MTT assay.

#### 2.3.1 Cell viability assay (MTT)

Cell viability was evaluated using the MTT assay. MTT (3-[4,5-dimethylthiazol-2-yl]-2,5-diphenyltetrazolium bromide) was prepared at 5 mg/mL in PBS and diluted in DMEM. A volume of 170 μL/well (150 μL DMEM + 20 μL MTT) was added to each well. Plates were incubated for 4 h at 37 °C with 5% CO_2_. Following incubation, the medium was removed, and 100 μL/well of dimethyl sulfoxide (DMSO) was added to dissolve the formazan crystals. Optical density was measured using a BioTek microplate reader (BioTek, Japan) at 540 nm.

#### 2.3.2 Morphological analysis

For morphological analysis, after 30 min of exposure to PAW or control medium, cells were fixed with 4% paraformaldehyde for 15 min at room temperature. Wells were washed three times with sterile PBS and incubated for 45 min in permeabilization/blocking solution (PBS containing 2% BSA and 0.5% Triton X-100), protected from light. F-actin filaments were stained with phalloidin (Invitrogen, United States), diluted 1:2,000 in PBS with 1% BSA (200 μL/well), and incubated for 2 h at room temperature. After staining, wells were washed three times with PBS, and nuclei were counterstained with DAPI. Imaging was performed at the National Institute of Photonics Applied to Cellular Biology (INFABIC, UNICAMP) using an EVOS M5000 Imaging System (Thermo Fisher Scientific) with a 20 × objective. Images were processed using ImageJ FIJI software with brightness and contrast adjustments.

### 2.4 Electrical characterization

The plasma reactor was powered by a sinusoidal high-voltage waveform with a peak-to-peak amplitude of approximately 8–9 kV. The applied frequency was varied in the range of 20–90 kHz to investigate frequency-dependent effects on power dissipation. Voltage and current measurements were performed using a two-channel digital oscilloscope (Rigol DS1074). Applied voltage was monitored with a high-voltage probe (Cal Test CT4028, 1000:1 attenuation ratio), while plasma current was obtained indirectly by measuring the voltage drop across a 10 Ω resistor connected in series with the grounded electrode. Current was calculated using Ohm’s law (I = *V*_*resistor*_ / R), where *V*_*resistor*_ is the measured voltage drop across the resistor and R = 10 Ω. Root mean square (RMS) power was determined using the following expression:


$$P=V_{\mathrm{rms}}\cdot I_{\mathrm{rms}}\,\cos\,\left(\varphi \right)$$

Where *V*_*rms*_ and *I*_*rms*_ are the RMS values of voltage and current, respectively, and φ is the phase angle between voltage and current.

## 3 Results and discussion

### 3.1 Electrical characterization

**FIGURE 2:**
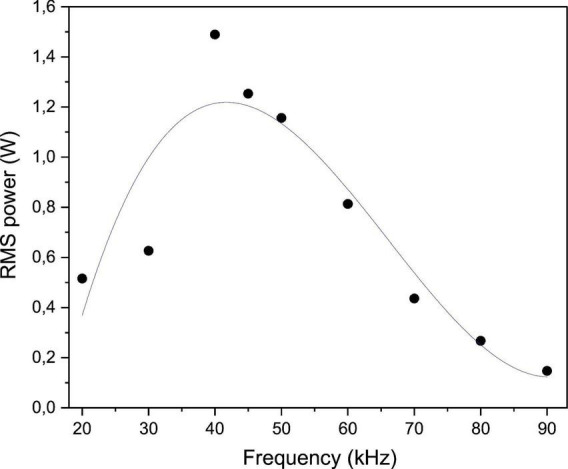
RMS power of the plasma as a function of applied voltage frequency in the range of 20–90 kHz.

Figure 2 presents the RMS power as a function of applied frequency in the range of 20–90 kHz. The results reveal a clear frequency-dependent behavior, with a pronounced peak in power observed in the 40–50 kHz range, reaching a maximum of approximately 1.5 W, followed by a progressive decline at higher frequencies. This behavior indicates the presence of a resonance-like condition in the 40–50 kHz region, where the electrical coupling between the power supply and the plasma is maximized, leading to more efficient energy transfer to the discharge.

Similar frequency-dependent trends in plasma power have been reported in previous studies, where the optimal operating frequency was associated with improved plasma stability and higher densities of reactive species (Abourayana et al., 2017; Litch et al., 2025; Rauner et al., 2019; Rusu and Cormier, 2003). At resonance conditions, the impedance of the plasma system is minimized, which results in higher current flow for a given applied voltage and, consequently, higher power deposition (Czarnetzki et al., 2006; Zhou et al., 2024).

On the other hand, at lower (20 kHz) and higher frequencies ( > 70 kHz), the reduced RMS power (0.15–0.63 W) suggests less efficient coupling of electrical energy into the plasma, possibly due to increased impedance mismatch between the power supply and the plasma load. To investigate whether this reduction in power deposition influences the physicochemical properties of plasma-activated water, we measured pH, oxidation-reduction potential (ORP), electrical conductivity, and total dissolved solids (TDS) across the tested frequency range (section 3.2).

Overall, these results emphasize the critical role of frequency tuning in plasma systems. The pronounced power absorption observed in the 40–50 kHz range, with a broad maximum reaching approximately 1.5 W, provides a solid rationale for selecting 40 kHz as the operating condition for subsequent physicochemical and biological analyses. This frequency was chosen because it lies within the region of maximum power deposition while ensuring stable and reproducible plasma discharge characteristics. The influence of this power deposition on water chemistry and antimicrobial efficacy is discussed in sections 3.2 and 3.3, respectively.

### 3.2 Effect of activation time and frequency on pH, ORP, TDS, and conductivity

**FIGURE 3:**
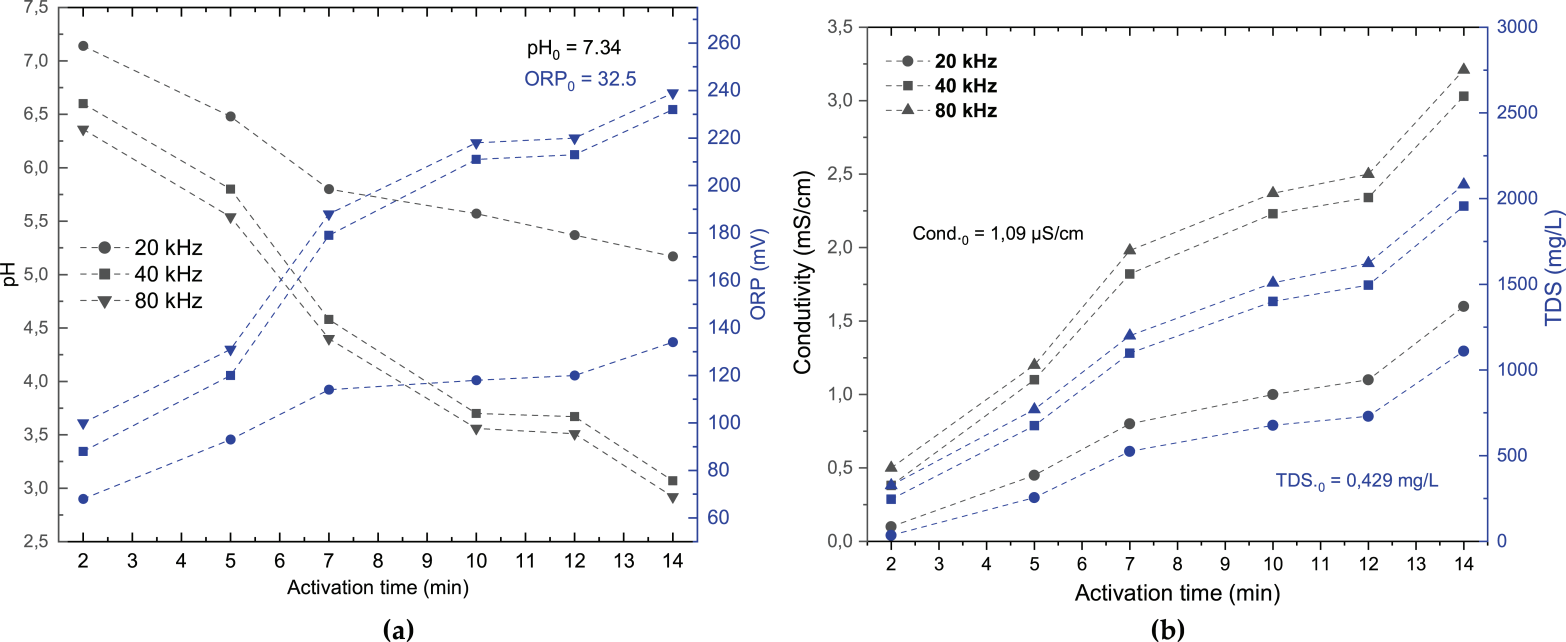
pH and ORP values **(a)**, as well as conductivity and TDS **(b)**, for PAW generated at different frequencies (20, 40, and 80 kHz) over activation times of up to 14 min. Black lines represent pH and conductivity (left axes); blue lines represent ORP and TDS (right axes). Different symbols and line styles distinguish the three operating frequencies. Data represent mean values from three independent experiments (pH: ± 0.02–0.03; ORP: ± 0.2–0.5 mV; conductivity: ± 1–2%; TDS: ± 0.04–0.06 mg⋅L^−1^).

The physicochemical characterization of PAW is essential to understanding the modifications induced by the reactive species generated during treatment. In the present study, the parameters of pH, conductivity, TDS and ORP were monitored during water to activation, as shown in Figure 3. Analyses were conducted at three different operating frequencies (20, 40, and 80 kHz) for up to 14 min of treatment, with the aim of identifying the frequency that yielded the most significant changes for subsequent time-dependent investigations.

Activated deionized water exhibited a substantial decrease in pH across all frequencies as the activation time increased, indicating significant acidification (Figure 3a). The initial pH of the deionized water was 7.34 ± 0.02. It is worth noting that after 14 min of activation, the pH values at 20, 40, and 80 kHz decreased to 5.09, 3.07, and 2.93, respectively. Acidification was less pronounced at 20 kHz, while both 40 and 80 kHz resulted in similar and more substantial pH reductions. A sharper pH decline was particularly evident after 7 min at higher frequencies. Previous studies have reported comparable reductions in water pH following plasma discharge treatment (Green et al., 2025; Kooshki et al., 2024; Kostya et al., 2020) often requiring longer treatment times to achieve significant acidification and microbial effects. Additionally, differences between stirred and static conditions during plasma treatment have been observed (Green et al., 2025; Hoeben et al., 2019) with static conditions exhibiting slower pH changes due to limited RONS diffusion. For instance, one study reported a pH drop below 3 after 20 min of DBD treatment in 1 liter of circulating tap water, attributed to improved reactive diffusion of reactive species (Kooshki et al., 2023).

The marked reduction in pH may enhance the antimicrobial efficacy of PAW through several mechanisms, including the increased stability and activity of RONS. In acidic environments, oxidizing compounds such as H_2_O_2_ remain more stable, promoting oxidative attacks against microbial cells (Deng et al., 2025; Naïtali et al., 2010). Furthermore, at low pH, NO_2_^–^ is converted into HNO, an unstable intermediate that can decompose into NO_3_^–^ and NO, the latter being a potent antimicrobial agent capable of diffusing across biological membranes and acting synergistically with H_2_O_2_ (Naïtali et al., 2010). This observation is also corroborated by previous studies attributing to the acidification of water by plasma is mainly due to the production of nitric acid from the NO produced by the plasma (Wu et al., 2017). In accordance with this, studies indicate that O_3_ generated during plasma treatment, promotes acidification upon dissolution in water, thereby enhancing the antimicrobial conditions of PAW and contributing to its high oxidation reduction potential (Perinban et al., 2022).

Similar to pH, ORP is a critical parameter for evaluating oxidative potential and electron transfer capacity in chemical systems (Wu et al., 2017). More recently, ORP has been widely adopted as one of the overall levels of ROS in the plasma-activated media (Xu et al., 2023). In contrast to the pH trend, ORP increased with activation time at all frequencies. After 14 min, ORP reached approximately 134.0, 232.0, and 239.0 mV for 20, 40, and 80 kHz, respectively. The 20 kHz frequency showed a gradual and less pronounced increase, while 40 kHz demonstrated a steeper rise after 7 min, peaking at 14 min. The behavior at 80 kHz was similar, with final values slightly higher but not markedly different from 40 kHz. ORP elevation is attributed to the accumulation of oxidizing species such as OH radicals, O_3_, H_2_O_2_, and HNO_2_ in the solution, which collectively enhance the antimicrobial potential of PAW (Bai et al., 2020; Copeland and Lytle, 2014; Lee et al., 2022; Tian et al., 2015).

Conductivity was also observed over time following PAW activation (Figure 3b), which suggests that the increase in conductivity is related to the presence of active ions generated in the plasma-liquid interaction process (Patra et al., 2022; Wu et al., 2017). After 14 min, conductivity remained lower at 20 kHz, while 40 and 80 kHz showed a more pronounced and similar linear rise. Wu et al., observed a comparable sharp increase in PAW conductivity, associated with ion accumulation from reactions between water molecules and plasma electrons, with also correlated with rising H_2_O_2_ concentrations and microbial inactivation potential (Wu et al., 2017).

Meanwhile, TDS increased with activation time across all frequencies. The most notable increases were observed at 40 and 80 kHz, while 20 kHz showed slight fluctuations and lower values overall. This trend likely results from the dissolution and accumulation of RONS in the solution (Pal et al., 2024)

Therefore, the comparative analysis of the three tested frequencies (20, 40 and 80 kHz) showed that both 40 and 80 kHz induced more pronounced physicochemical changes, including greater acidification, higher ORP and increased conductivity, suggesting a more effective generation and accumulation of reactive species under these conditions. In contrast, the lower frequency of 20 kHz resulted in milder shifts in these parameters, particularly showing limited pH reduction and lower ORP values, which may not be sufficient to support subsequent microbial inactivation treatments. Similar results were reported by Asimakopoulou et al. (2022) and Liao et al. (2017) where higher frequencies combined with sufficient exposure time resulted in stronger oxidative conditions in plasma-activated media. Although 80 kHz showed slightly higher ORP and conductivity values, the differences compared to 40 kHz were minimal, which aligns with studies suggesting that beyond a certain frequency threshold, further increases do not significantly enhance PAW reactivity. This plateau effect has been frequently described in the literature (Litch et al., 2025; Rauner et al., 2019; Xu et al., 2011).

Based on these findings, 40 kHz was selected for subsequent analyses as a function of activation time. It is important to note that the physicochemical properties of PAW, such as pH, ORP, and conductivity are closely linked to the presence of RONS in water, which directly influences its antimicrobial performance (Bai et al., 2020; Deng et al., 2025; Lee et al., 2022; Perinban et al., 2022; Sampaio et al., 2022; Tian et al., 2015; Wu et al., 2017). Accordingly, the following sections explore how activation time affects the formation of specific reactive species (NO_2_^–^, NO_3_^–^, H_2_O_2_, O_3_, and HNO_2_), using UV-Vis spectroscopy to better understand their role in the physicochemical transformations observed.

### 3.3 UV−Vis spectrophotometry analysis

**FIGURE 4:**
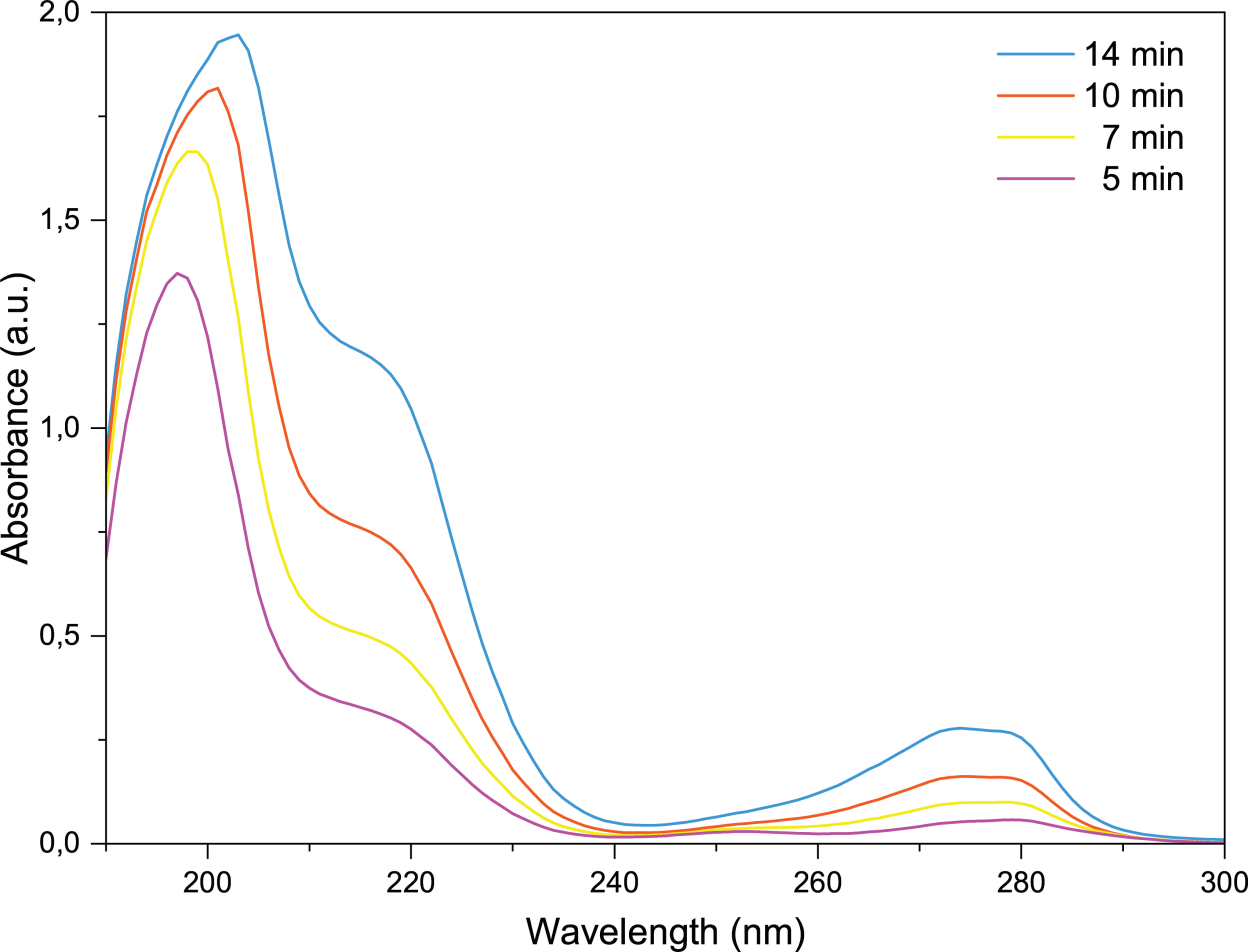
UV-Vis absorption spectra of plasma-activated water (PAW) obtained after different activation times (5, 7, 10, and 14 min) for qualitative identification of dissolved RONS.

Figure 4 shows the UV-Vis absorbance spectra of PAW produced by DBD air plasma bubbling after exposure times of 5, 7, 10, and 14 min. It is evident that an increase in activation time is accompanied by a broadening and intensification of the spectra, as indicated by the changes in the absorption characteristics. While the spectral profiles remained generally similar, distinct differences in intensity and width emerged with longer plasma exposures. A broad absorption band appeared between approximately 190 and 240 nm, whose intensity and width progressively increased with prolonged treatment, becoming particularly prominent after 10 min. This region is associated with the presence of reactive RONS in PAW, as reported in previous studies (Chiappim et al., 2021; Oh et al., 2018). At 14 min, the absorbance maximum near 200 nm was nearly twice as high as that observed at shorter activation times, indicating the cumulative generation and accumulation of plasma-derived species in the aqueous phase. The spectra demonstrate that plasma activation induces significant chemical changes in the solution, resulting in the formation of various species with characteristic absorption features in this region, and showing a clear time-dependent trend (Oh et al., 2018; Szili et al., 2015).

While the intrinsic absorbance profile of pure water is well characterized (Pope and Fry, 1997), the spectra of PAW are markedly more complex due to the presence of RONS, each contributing unique absorption features. In the context of gas phase ROS generation, the choice of working gas plays a pivotal role in shaping the chemical composition of the resulting PAW. For instance, plasmas generated in inert gases such as Ar or He predominantly yield H_2_ as the primary neutral species due to negligible oxygen and nitrogen contents (Mohades et al., 2020). This variation in gas composition directly influences the formation of various RONS, affecting both the reaction pathways and the overall oxidative potential of the plasma system. In other words, the nature and abundance of these species are strongly dependent on the working gas composition, as extensively reported in previous studies (Machala et al., 2018; Mohades et al., 2020).

In particular, when compressed air is used, O_3_ typically predominates among the generated species, due to the high oxygen content (∼21%) in atmospheric air, which favors ozone formation under plasma excitation (Angelina Cullen et al., 2025). The spectral region between 240 and 280 nm highlights the characteristic absorption of O_3_, as previously reported and consistent with findings from ozonated water studies (Miranda et al., 2024; Oh et al., 2016b). The marked increase in absorbance within this region, especially after 10 and 14 min of plasma exposure, supports the progressive enrichment of O_3_ in the PAW which can be attributed to the composition of air and the abundant oxygen in it.

The broadening of this band may also reflect increased stabilization of ozone in solutions, potentially facilitated by pH changes or by the generation of secondary species that inhibit ozone decomposition (Biń, 2006). Additionally, the gradual broadening observed in this spectral region highlights the dynamic interaction between ozone and other reactive oxygen species (ROS), such as H_2_O_2_ and •OH, which may coexist and interact synergistically (Oh et al., 2016b; Pavlovich et al., 2013).

Among the possible mechanisms involved, the role of excited oxygen atoms (O) is particularly significant, as discussed by Czarnetzki et al. (2006), Benedikt et al. (2018), and Liu et al. (2015). These highly reactive species contribute to the formation of secondary oxidizing agents. For example, O* (Reaction 1) can react directly with water molecules to form H_2_O_2_ (Liu et al., 2015), or with dissolved oxygen to generate O_3_ (Reaction 2) (Benedikt et al., 2018) . Such reactions, together with the direct solubilization of O_2_ and O_3_ into the aqueous phase, contribute substantially to the chemical complexity of the treated water:


$$\left(1\right)\to O*+H_{2}O\to H_{2}O_{2}$$

$$\left(2\right)\to O*+O_{2}\left(aq.\right)\to O_{3}$$

The continuous increase in absorbance between 250 and 300 nm provides compelling evidence for the role of ozone in PAW chemistry. A strong correlation between O_2_ and O_3_ has been reported, where a high concentration of dissolved O_2_ favors the formation of O_3_ via the reaction (Murray et al., 2022). The maximum concentration of O_3_ (1.38 mg/L) was measured after 14 min of treatment while the H_2_O_2_ concentration was found to be more than twice as high. This suggests that, given the elevated relative concentration of H_2_O_2_, the reaction (CDC, 2019) is likely to represent the predominant formation pathway for reactive species, owing to the abundant availability of water molecules. Moreover, the broadening of the absorption band between 190 and 240 nm with prolonged activation times can be attributed to the increasing concentrations of H_2_O_2_, •OH, and primary RNS.

**FIGURE 5:**
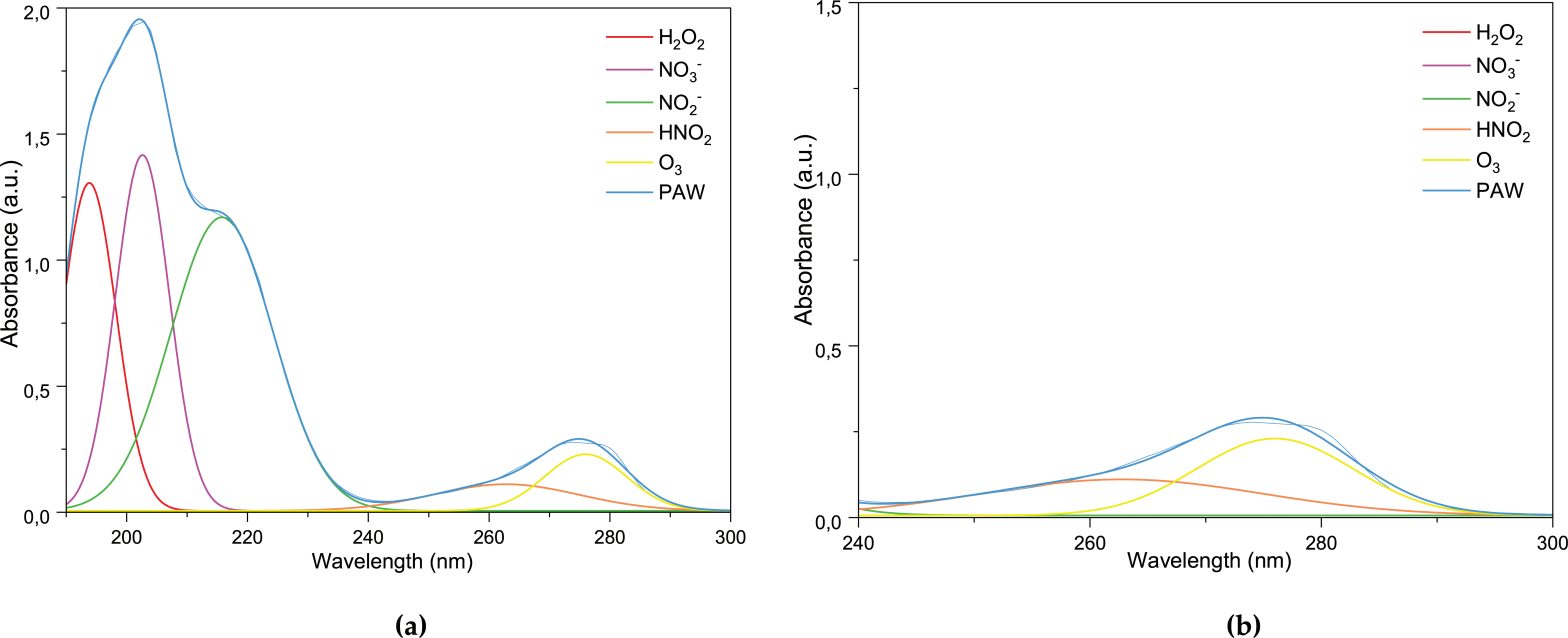
Deconvolution of UV-Vis absorption spectra of PAW and reference reactive species for qualitative identification of dissolved RONS. **(a)** Full spectrum (200–300 nm) showing characteristic absorption peaks of H_2_O_2_, NO_3_^–^, NO_2_^–^, HNO_2_, and O_3_. **(b)** Focus on the 240–300 nm region highlighting the spectral contributions of O_3_ and H_2_O_2_ relative to the PAW matrix.

In Figure 5 one can see the deconvolution of the spectra recorded after activation for 14 min. The deconvolution method was employed to pinpoint the presence of reactive species in the PAWs (Oh et al., 2018). The complexity of the UV absorbance spectrum of PAW, along with the potential spectral overlap, highlights the multifaceted nature of the reactions occurring during plasma treatment. These interactions may also lead to the formation of intermediate compounds with overlapping absorbance, further contributing to the spectral complexity through multiple absorption peaks associated with electronic transitions. Therefore, the absorbance spectra were deconvoluted to qualitatively identify the presence of NO_2_^–^, NO_3_, H_2_O_2_, HNO_2_, and O_3_ (Liu et al., 2019; Szili et al., 2015).

The peaks at approximately 190 and 195 nm are associated with H_2_O_2_ and dissolved oxygen, respectively. An increase in the level of dissolved oxygen has been shown to enhance total absorption in the 190–210 nm range. This assignment is supported by the well-documented spectral characteristics of H_2_O_2_ in aqueous solutions, which exhibit a prominent band in this range due to electronic transitions (Liu et al., 2019; Mohades et al., 2020; Oh et al., 2016a; Poggemann et al., 2025). Moreover, the presence of H_2_O_2_ in the plasma-activated solution was confirmed through quantification and the broadening of the absorption spectra with prolonged activation times correlates with increasing H_2_O_2_ concentration, as show in Table 1 for 7 and 14 min. Among the proposed processes, the recombination of dissolved •OH is a widely accepted pathway for H_2_O_2_ formation (Chen et al., 2022; Kostya et al., 2020):


$$OH\cdot +OH\cdot \to H_{2}O_{2}\;\left(3\right)$$

$$O\,H+H\,O_{2}\to H_{2}\,O_{2}+O_{2}\;\left(4\right)$$

**TABLE 1:** H_2_O_2_, O_3_, HNO_2_, NO_2_^–^, and NO_3_^–^ concentrations after PAW for 7 and 14 min.

Time	H_2_O_2_ (mg/L)	O_3_ (mg/L)	HNO_2_ (mg/L)	NO_2_^–^ (mg/L)	NO_3_^–^ (mg/L)
7 min	1.57	0.82	0.074	1.0	10.0
14 min	2.71	1.38	10.17	5.0	50.0

These reactions occur on a microsecond timescale and predominance under conditions of elevated OH concentration, particularly in acidic media where the hydroperoxyl radical (HO_2_•) also contributes to H_2_O_2_ formation (Tampieri et al., 2021).

As shown in Table 1, the H_2_O_2_ strip tests indicated a gradual increase in H_2_O_2_ concentration, ranging from 1.5 to 2.7 mg/L over 7 to 14 min of activation. These results suggest that the direct bubbling configuration adopted in this study provides favorable conditions for the transfer and solubilization of plasma-generated reactive species into the aqueous phase. The bubbling configuration optimizes RONS transfer through dual mechanisms: plasma-generated species are rapidly transported by the air flow directly into the liquid phase, minimizing gas-phase recombination losses, while the small bubble size maximizes the gas-liquid interfacial area, enhancing dissolution efficiency of reactive species. The associated turbulence further promotes solution homogeneity.

Although some studies have focused on plasma jet treatment applied directly to the liquid surface, where increased humidity and UV radiation contribute to the generation of OH• and H_2_O_2_, the bubbling approach also enables efficient mass transfer from the gas phase. In this system, the higher concentrations of H_2_O_2_ can be attributed to multiple contributing mechanisms, including the recombination of •OH generated in the plasma and dissolved in the liquid, as well as potential UV-induced photolysis at the gas–liquid interface. While the extent of UV penetration is limited in direct bubbling systems, interactions at the interface likely play a role in the observed accumulation of H_2_O_2_ over time (Huang et al., 2024; Kim et al., 2014; Kostya et al., 2020; Oh et al., 2018).

Subsequent to the detection of hydrogen peroxide, the UV spectra also revealed the presence of nitrogen-derived species. A prominent absorption peak was observed near 200 nm, attributed to NO_3_^–^, while a second, smaller peak appearing at a slightly longer wavelength corresponds to NO_2_^–^. These results are consistent with previous reports indicating that NO_3_^–^ tends to be more abundant than NO_2_^–^ in air and oxygen plasmas. Both NO_2_^–^ and NO_3_^–^ are short-lived ions in the gas phase, undergoing rapid formation and consumption (Ghorui et al., 2024; Spicer et al., 1993). Notably, density functional theory studies support the assignment of the NO_3_^–^ peak at 200 nm, highlighting a strong π→π* transition responsible for its optical absorption in this region (Madsen et al., 2003; Pedersen et al., 2019).

Activation time has been shown to have significant impact on NO_2_^–^ and NO_3_^–^ concentrations. However, the concentrations of NO_2_ and NO_3_ were found at lower rates when compared to H_2_O_2_ and O_3_. The formation of H_2_O_2_ and O_3_ occurs through fast, direct reactions involving reactive oxygen species generated from water or oxygen dissociation in the plasma. In contrast, the formation of NO_2_^–^ and NO_3_^–^ requires multiple steps, including gas phase nitrogen oxide generation followed by dissolution into the liquid, making this process less efficient and more condition-dependent (Pandey et al., 2023; Xu et al., 2023).

Another important consideration is that the higher concentrations of H_2_O_2_ observed in this work may be related to its interaction with NO_2_^–^. As reported in previous studies using different gases and discharge modes, the formation of peroxynitrous acid (OONOH⋅H2O) consumes both H_2_O_2_ and NO_2_^–^. Therefore, reduced NO_2_^–^ availability limits this reaction, resulting in higher residual H_2_O_2_ concentrations (Milhan et al., 2022; Pandey et al., 2023).

In plasma systems, other gases predominantly generate H_2_ as the main neutral species in the gas phase, while in compressed air, O_2_ emerges as dominant species in the gas phase (Yuan et al., 2018). Due to their low Henry’s law constants, these species tend to remain in the gas phase. When considering RNS in the liquid phase, OH has a transient presence in both the gas and liquid phases (Gorbanev et al., 2016; Liu et al., 2019). Aqueous OH is produced through several mechanisms as previously discussed. As Lietz and Kushner (2016) highlighted, an increase in pulse frequency in DBD plasmas reduces the density of most RONS in the liquid due to their propensity to react with other RONS in the gas phase prior to solvation. Therefore, notable exceptions to this observation are aqueous O_3_, which has demonstrated as precursor or gas phase, and H_2_O_2_, due to the recombination products being more stable, thus benefiting from a higher repetition rate (Chiappim et al., 2021; Milhan et al., 2022). This hypothesis is consistent with the low concentrations of NO_3_^–^ and NO_2_^–^ observed in this study.

This behavior can also be explained by the higher energy required for N_2_ dissociation compared to O_2_. Nitrogen has a stronger triple bond, making its dissociation less efficient under the same plasma conditions. As a result, there is preferential generation of atomic oxygen (O), which favors the formation of ROS, such as O_3_ and H_2_O_2_, over nitrogen-based species like NO, NO_2_^–^, and NO_3_^–^. This observation aligns with previous studies that described discrete discharge modes in air plasma systems, specifically the O_3_-rich mode and the NO_x_-rich mode (Xu et al., 2023). The present system appears to operate predominantly in the O_3_ mode, favoring the production of ozone-enriched gas while suppressing nitrogen oxide generation.

In Figure 5, two partially overlapping peaks are observed, both contributing to the shape and intensity of the 240–300 nm envelope. In particular, the gradual band broadening and the appearance of a shoulder around 260–300 nm in the deconvolved spectra can be attributed to the presence of HNO_2_ (López Pasquali et al., 2007). Notably, the intensity of this band increases after 10 min of activation, which correlates with a decrease in pH during the treatment time, favoring HNO_2_ formation (Arakaki et al., 1999). In more acidic environments, as observed (pH 3.07), the higher concentration of H^+^ favors the reaction: *H*^+^+*NO*2-→*HNO*2. This leads to the appearance of a discrete absorption band attributed to this species. Although the quantified concentration of NO_2_^–^ was low (5 mg/L), some of it may have been consumed to form HNO_2_, contributing to the observed absorption. Based on the acid-base equilibrium, the maximum concentration of HNO_2_ in the sample was estimated to be around 10.17 mg/L at 14 min (Table 1), confirming its detection despite its instability potential and partial degradation by strong oxidants such as ozone and hydrogen peroxide (Yuan et al., 2025).

In summary, the absorbance spectra indicates that the treatment of PAW induces significant chemical changes that are dependent on treatment duration, likely influences the concentration of reactive species and, consequently, the antimicrobial potential of the solution. As previously reported, microbial inhibition processes by PAW are often associated with the presence of RONS, such H_2_O_2_, NO_3_^–^, HNO_2_, NO_2_^–^ with their combined concentrations playing an important role in the biological effects. In this present investigation, to understand the relevant and combined presence of ROS species, such as O_3_ and H_2_O_2_, may play a predominant role in shaping the antimicrobial activity, particularly in contrast to nitrogen-derived species, which were detected at lower levels (Fateev et al., 2006; Oh et al., 2016b; Pavlovich et al., 2013).

The system under scrutiny was shown to promote the formation of O_3_ and H_2_O_2_ while concurrently producing diminished levels of NO_3_^–^ and NO_2_^–^. These findings characterize the distinct chemical composition of PAW produced in this study and establish a basis for subsequent biological evaluations aimed at correlating the reactive species profile with antimicrobial efficacy.

### 3.4 Microbiological assays

The microbiological assessment of [PAW was conducted to investigate its antimicrobial potential against clinically and hygienically relevant microorganisms under two distinct exposure and activation times, as described in the Materials and methods section. The study included two bacterial strains, *Staphylococcus aureus* (Gram-positive) and *Escherichia coli* (Gram-negative), as well as the opportunistic fungus *Candida albicans.* These species are commonly employed in antimicrobial testing due to their clinical relevance and the growing concern regarding resistance to conventional therapies (Han et al., 2022; Sharma et al., 2024; Shen et al., 2016).

**FIGURE 6:**
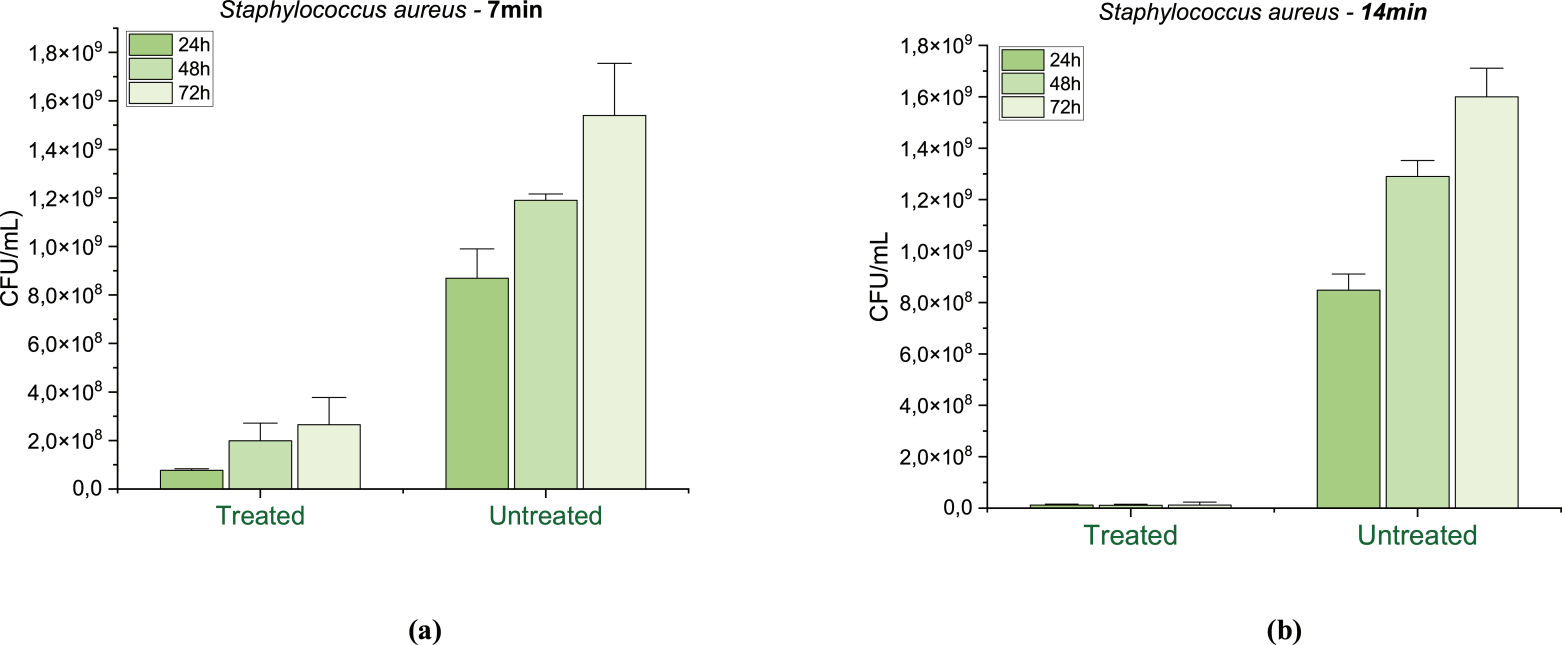
Mean and standard deviation of bacterial growth inhibition for Staphylococcus aureus after **(a)** 7 min and **(b)** 14 min of PAW treatment, evaluated at 24, 48, and 72 h. Control: untreated group. Statistically significant differences between treated and control groups were observed at all points (*p* < 0.01), according to one-way ANOVA followed by Tukey’s *post-hoc* test (*n* = 3).

Figure 6a illustrates the effect of 7-min PAW treatment on *Staphylococcus aureus*, showing a substantial inhibition of microbial growth. Growth was inhibited by 91.17% at 24 h (*p* = 0.0076), 83.31% at 48 h (*p* = 0.0006), and 82.73% at 72 h (*p* = 0.0028), compared to the untreated control. These results suggest a predominantly bacteriostatic effect under this activation and exposure time. In contrast, Figure 6b shows that extending the PAW exposure to 14 min significantly enhanced the antimicrobial activity, with mean inhibition rates of 99.15% at 24 h (*p* = 0.0018), 99.13% at 48 h (*p* = 0.0008), and 99.25% at 72 h (*p* = 0.0014), indicating a potential bactericidal outcome associated with longer plasma activation.

These findings are consistent with previous studies using PAW generated by different plasma systems. For instance, PAW generated via plasma bubbling has been show to achieve 3–4 log reduction in *Staphylococcus aureus* viability, with antimicrobial efficacy attributed to the presence of RONS, which cause damage to cell membranes and intracellular structures. In a study conducted by Zhang et al. (2017), atmospheric helium dielectric barrier discharge was applied in a gas–liquid interface to inactivate S. *aureus*, with bacterial inactivation increased with treatment time, reaching 9.3, 37.2, 81.8, and 86.7% after 1, 3, 5, and 8 min of exposure, respectively. Xu et al (2020) also reported a significant reduction and inhibition biofilm regeneration in S. *aureus* after treatment with PAW produced by sliding arc system using compressed air. Likewise, Chiappim et al. (2021) demonstrated a 3-log reduction in S. *aureus* viability after 10 min of PAW activation. This effect was linked to the low pH (∼3.5) and high ORP, parameters that are comparable to those observed in the present study, further supporting the role of RONS accumulation as a key antimicrobial mechanism.

**FIGURE 7:**
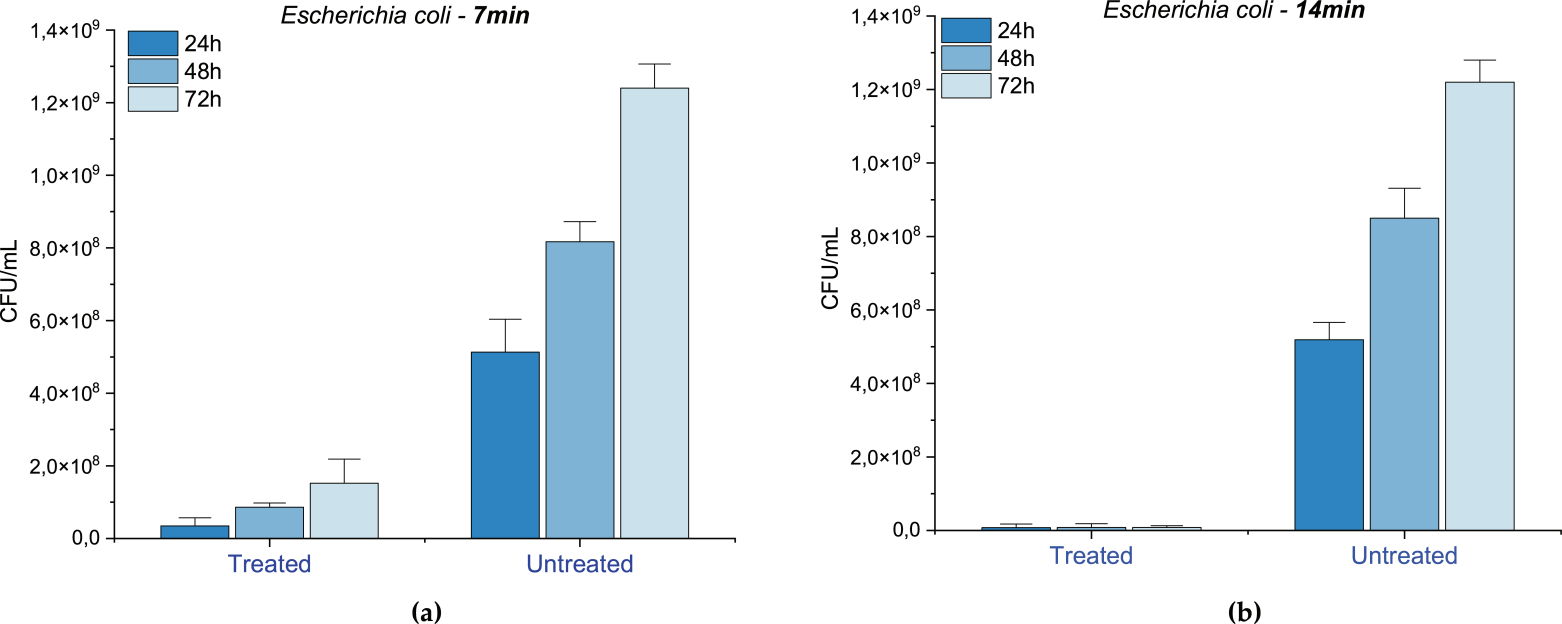
Mean and standard deviation of bacterial growth inhibition for Escherichia coli after **(a)** 7 min and **(b)** 14 min of PAW treatment, evaluated at 24, 48, and 72 h. Control: untreated group. Statistically significant differences between treated and control groups were observed at all-time points (*p* < 0.01), according to one-way ANOVA followed by Tukey’s post-hoc test (*n* = 3).

PAW treatment also proved effective against *Escherichia coli* as shown in Figure 7. Seven minute exposure against inhibited bacterial growth by 93.30% after 24 h (*p* = 0.0086), 89.47% after 48 h (*p* = 0.0028), and 87.76% (*p* = 0.0006) after 72 h, suggesting the same bacteriostatic effect previously observed at this activation time (Figure 7a). Conversely, the 14-minute treatment (Figure 7b) resulted in inhibition rates of 99.68% at 24 h (*p* = 0.0027), 99.47% at 48 h (*p* = 0.0029), and 99.55% at 72 h (*p* = 0.0007), reflecting a pronounced bactericidal effect and reinforcing the efficacy bacterial effect and of prolonged PAW activation and exposure.

Other studies supported the influence of increased treatment time on E. *coli* inhibition. For instance, PAW generated by plasma jets achieved reductions of 0.41, 0.64, and 1.18 log CFU/mL after 15, 30, and 60 min, respectively, indicating a reduction of up to 98.5% in bacterial viability, with SEM images showing visible membrane damage (Sawangrat et al., 2022). Furthermore, Traylor et al. (2011) observed a significant reduction in E. coli after exposure to PAW generated for 15 by air dielectric barrier discharge. In another study, atmospheric micro-plasmas using different gases demonstrated that longer treatment times enhanced bacterial inactivation due to increased generation of reactive oxygen species. Specifically, compressed air was more effective than other gases, supporting its potential as a feed gas for PAW production to inactivate *E. coli* (Zhou et al., 2015).

**FIGURE 8:**
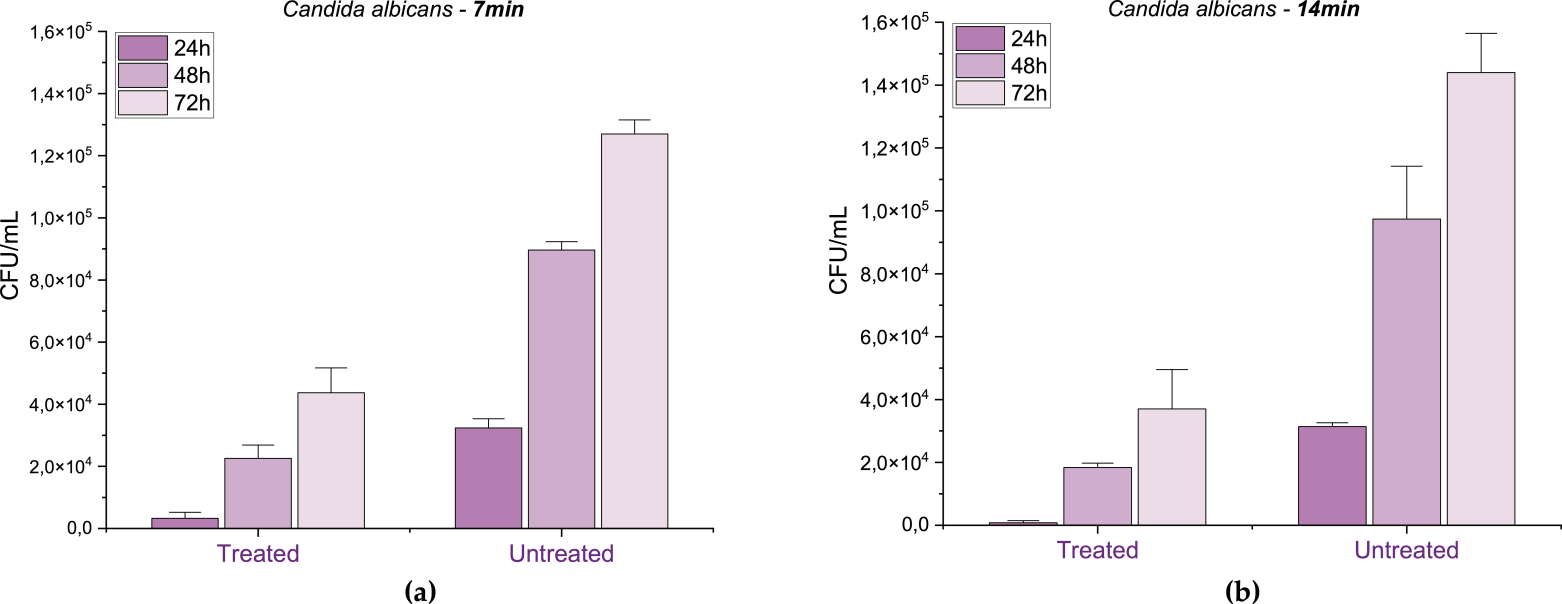
Mean and standard deviation of fungal growth inhibition for Candida albicans after **(a)** 7 min and **(b)** 14 min of PAW treatment, evaluated at 24, 48, and 72 h. Control: untreated group. Statistically significant differences between treated and control groups were observed at all points (*p* < 0.01), according to one-way ANOVA followed by Tukey’s *post-hoc* test (*n* = 3).

Regarding *C. albicans*, the PAW treatment for 7 min (Figure 8a) resulted in growth inhibition of 88.45% at 24 h (*p* = 0.00035), 73.82% at 48 h (*p* = 0.00008) and 65.68% at 72 h (*p* = 0.00041), suggesting a strong initial fungistatic effect that diminished over time, possibly due to the depletion of reactive species or microbial adaptation. When the exposure was extended to 14 min (Figure 8b), growth inhibition 97.45% at 24 h (*p* = 0.00003), 81.12% at 48 h (*p* = 0.01421) and 74.28% at 72 h (*p* = 0.00141). This pattern, characterized by high initial inhibition followed by partial recovery, is characteristic of a fungistatic rather than a fungicidal effect, suggesting that PAW temporarily interfered with the metabolism and proliferation of *C. albicans*, without leading to complete cell elimination.

Previous studies support this interpretation. Chiappim et al. (2021) investigated the antimicrobial activity of PAW generated by a sliding arc plasma system and found that, while it effectively inactivated bacteria, its action against C. *albicans* was limited to growth inhibition rather than complete elimination. Similarly, a study using a coaxial DBD reactor found a 37% reduction in *C. albicans* viability after 30 min of PAW exposure (Miranda et al., 2023). Additionally, other non-thermal plasma studies showed that while PAW can inactivate planktonic Candida cells and enhance antifungal susceptibility, its effect on Candida biofilms remains limited, reinforcing the view of a fungistatic mode of action (Sun et al., 2012)

PAW effectively inhibited the growth of both *S. aureus* and *E. coli*, with *S. aureus* exhibiting slightly lower sensitivity. This difference may be attributed to structural factors, as reactive species primarily target the outer membrane of Gram-negative bacteria, whereas in Gram-positive bacteria, they must penetrate a thicker peptidoglycan layer to reach intracellular components. Despite this biological distinction, statistical analysis confirmed that both organisms responded significantly and consistently to PAW treatment. The results demonstrated that activation time had a significant impact on bacterial inhibition, with 14-min treatments consistently yielding greater reduction rates than 7-min exposures (*p* < 0.0001 for both species). While shorter activation times led to partial inhibition (82–93%), extended exposure achieved reductions above 99% across all incubation periods, suggesting a transition from a predominantly bacteriostatic to a bactericidal effect.

The progressive increase in microbial inhibition observed with prolonged PAW activation supports the hypothesis that extended plasma treatment enhances the accumulation of RONS, leading to irreversible cellular damage. In contrast, 7-min activation resulted in only partial inhibition, indicating that the concentration of reactive species generated under these conditions was sufficient to disrupt cellular metabolism and division but insufficient to induce complete microbial inactivation. The 72-h assessment further reinforced the sustained antimicrobial activity of PAW under longer activation times, emphasizing its potential for long-term microbial control.

Compared to the bacterial strains, *Candida albicans* exhibited a distinct response profile. While PAW treatment was also effective in significantly reducing fungal growth at all-time points (*p* < 0.0001), the maximum inhibition observed was slightly lower than in bacteria, reaching 97.45% under 14-min activation. Notably, 7-min exposures led to strong initial inhibition, but a gradual loss of effectiveness was observed over time. This fungistatic pattern, with partial regrowth, is driven by the structural complexity of fungal cells: the robust cell wall rich in chitin and β-glucans provides superior barrier protection against oxidative stress (Chiappim et al., 2021; Brown et al., 2014), while efficient stress-response mechanisms, including antioxidant defenses and DNA repair systems (Chiappim et al., 2021). Our 97.45% inhibition at 14 min substantially exceeds the 37% reduction reported by Miranda et al. using 30-min PAW exposure and aligns with Chiappim et al.’s finding that PAW achieves growth inhibition rather than complete elimination of *C. albicans*. However, similar to other non-thermal plasma studies (McGain et al., 2017) partial regrowth indicates a fungistatic rather than fungicidal mode of action at shorter activation times. Statistical analysis confirmed that treatment, activation time, and incubation period all significantly influenced fungal viability, reinforcing the importance of optimizing plasma parameters for effective antifungal applications.

Additionally, the energy efficiency analysis at 24 h demonstrated that the system achieved remarkably high efficiencies ranging from 5.463 to 8.554 log/kWh (Table 2), with *E. coli* exhibiting the highest efficiency, followed by *S. aureus* and *C. albicans*. These values reflect the extremely low power consumption (1.25 W) required to achieve effective microbial inactivation. The superior performance against Gram-negative bacteria reflects their greater susceptibility to RONS-mediated oxidative damage, while lower efficiency for *C. albicans* aligns with fungal cell wall resistance and stress-response mechanisms. The use of compressed ambient air eliminates expensive noble gas requirements, significantly reducing operational costs.

**TABLE 2:** Energy efficiency of plasma-activated water antimicrobial activity at 24 h.

Microorganism	Activation time (min)	Inhibition (%)	Energy (kWh)	Efficiency (log/kWh)
*S. aureus*	7	91.17	0.000146	7.230
*S. aureus*	14	99.15	0.000292	7.100
*E. coli*	7	93.30	0.000146	8.053
*E. coli*	14	99.68	0.000292	8.554
*C. albicans*	7	88.45	0.000146	6.426
*C. albicans*	14	97.45	0.000292	5.463

Energy efficiency at 24 h, calculated as log reduction per kWh. Power: 1.25 W at 40 kHz.

Compared to conventional thermal disinfection, PAW offers substantial practical advantages. Steam sterilization consumes 1.9 kWh per kilogram of material sterilized (Lee et al., 2022), requires high temperatures (121–134°C), extended processing times, and is unsuitable for heat-sensitive materials. In contrast, PAW achieves effective microbial inactivation at room temperature with significantly lower energy input (0.000146–0.000292 kWh per treatment), representing an energy reduction of approximately 6.500-fold compared to thermal methods. This makes PAW particularly suitable for non-thermal treatment of liquids, heat-sensitive surfaces, and materials that cannot withstand high-temperature sterilization. These characteristics position compressed air-based PAW as an exceptionally energy-efficient and versatile antimicrobial technology.

### 3.5 Cytotoxicity and morphological response to PAW

Considering the potential of PAW as an antimicrobial agent, it is essential to ensure that its application does not pose a cytotoxic risk to target cells. The L929 cell line, which is derived from murine fibroblasts, was selected as a standard model for evaluating the toxicity of materials due to its broad acceptance in regulatory protocols (ISO 10993-5) for the evaluation of tissue compatibility. This supports the investigation of PAW as a safe alternative for potential biomedical applications.

**FIGURE 9:**
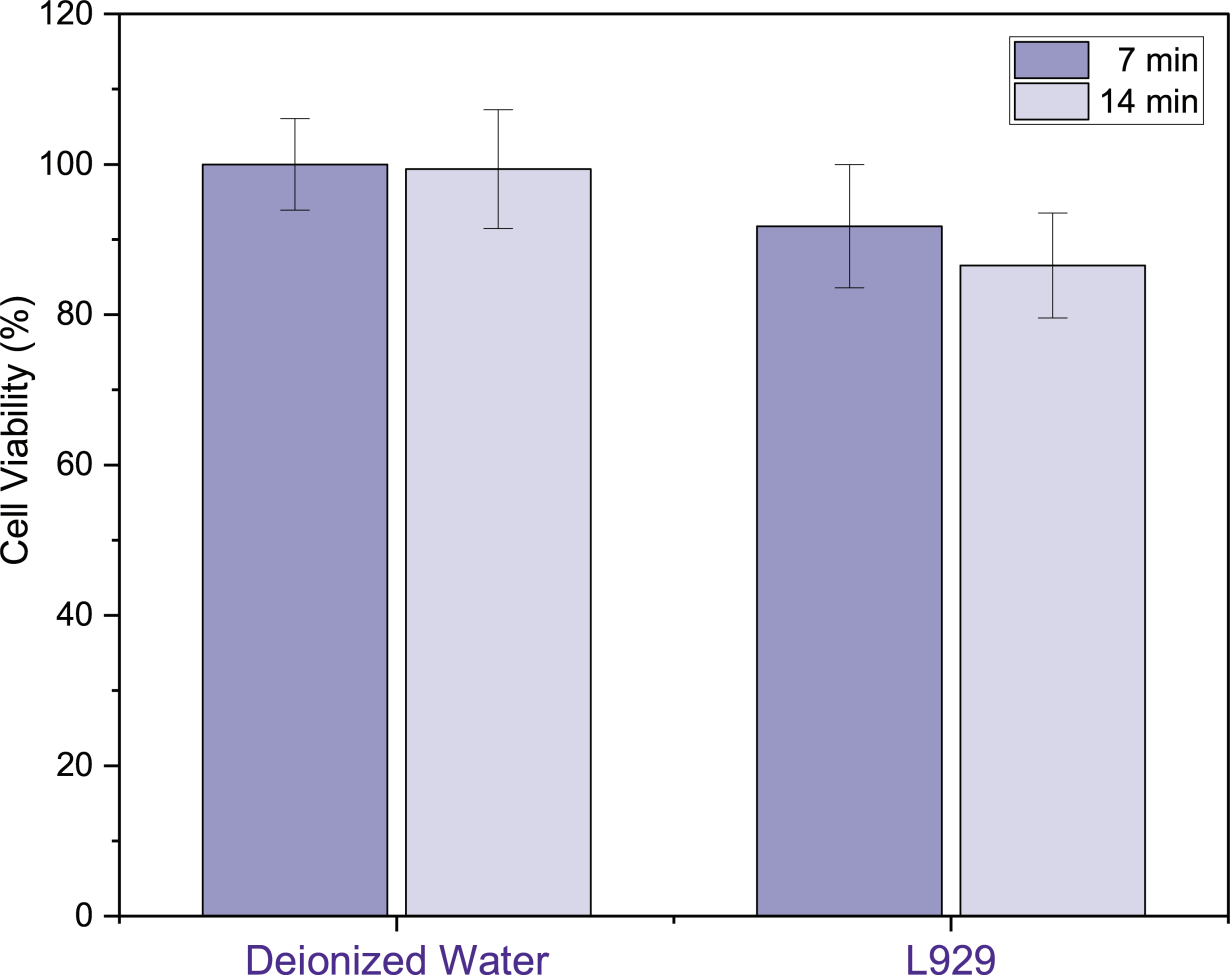
Viability of L929 fibroblasts after exposure to PAW activated for 7 and 14 min, compared to deionized water controls. Cells were exposed for 30 min, and viability was assessed by MTT after 24 h. All values remained above the 70% cytotoxicity threshold (ISO 10993–5). Data represents SD (*n* = 12); one-way ANOVA, *p* = 0.079.

Figure 9 presents the results of the cytotoxicity assessment of PAW on the L929 cell line. Cell viability was approximately 91.76 and 86.54% after 7- and 14-min activation, respectively, while the corresponding values for deionized water controls were 100% and 99.35%. All values remained above the 70% cytotoxicity threshold defined by ISO 10993-5, indicating no relevant cytotoxic effect. Statistical analysis using ANOVA and Kruskal–Wallis Tests confirmed that there were no significant differences between the experimental groups (p = 0.079), supporting the conclusion that PAW does not compromise the viability of L929 cells under the evaluated conditions.

Similarly, a previous study supporting the biocompatibility of plasma-treated solutions in fibroblasts. Horiba et al. (2017) reported that PAW generated by microwave plasma did not induce cytotoxicity in fibroblasts or keratinocytes after 24 h of exposure, with MTT and CCK-8 assays showing viability comparable to controls. Similarly, Ighodaro and Akinloye (2018) demonstrated that exposure to plasma-activated media provided cytoprotective effects in fibroblasts against H_2_O_2_-induced oxidative damage, via activation of the Nrf2-HO-1 antioxidant pathway. These findings suggest that, when properly controlled, plasma-treated solutions can enhance cellular resilience to oxidative stress. Consistently, in the present study, L929 fibroblasts remained above the cytotoxicity threshold even after exposure to PAW activated for 7 and 14 min.

To further characterize the cellular response to PAW, morphological analysis by fluorescence microscopy was performed. This approach allowed the visualization of potential structural alterations in both L929 fibroblasts and B16F10 melanoma cells. In the case of B16F10, the analysis was conducted in an exploratory manner to investigate potential selective effects of PAW.

**FIGURE 10:**
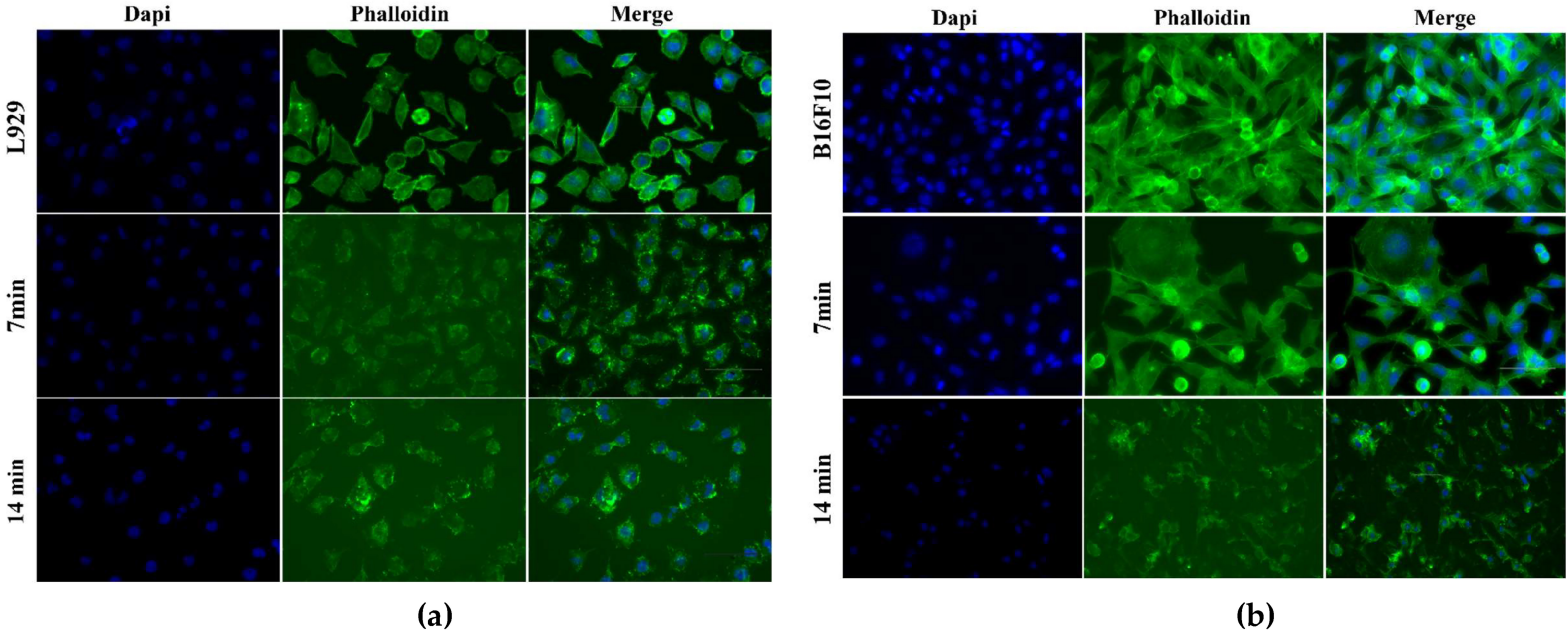
Fluorescence microscopy images of L929 fibroblasts **(a)** and B16F10 melanoma cells **(b)** after exposure to PAW activated for 7 and 14 min. Cells were stained with DAPI (blue) to visualize nuclei and with Phalloidin (green) to label the actin cytoskeleton.

As illustrated in Figure 10, the morphological analysis yielded additional information that served to further elucidate the cellular responses to PAW. In L929 fibroblasts, Figure 10a, although a slight reduction in fluorescence intensity and cytoskeletal organization was observed after PAW exposure, particularly at 14 min, the overall fibroblastic morphology, nuclear integrity, and cell confluency were largely preserved, which is consistent with the cell viability results. Interestingly, B16F10 melanoma cells exhibited more pronounced morphological alterations. After 7 min of PAW activation, changes in actin distribution and nuclear morphology were already apparent. These effects became more marked at 14 min, with visible evidence of cytoskeletal disorganization, cell rounding, reduced adherence, and lower cell density.

Studies indicate that the differential response between normal and tumor cells to PAW is closely linked to their distinct redox balance and antioxidant capacities. Normal cells possess enzymatic defenses such as superoxide dismutase, catalase, and glutathione peroxidase, which efficiently neutralize RONS (Estrella et al., 2013). In contrast, tumor cells, due to impaired redox regulation and elevated basal oxidative stress, are more susceptible to RONS accumulation and apoptosis induction (Jena et al., 2023).

Within this context, it is vital to understand how endogenous RONS are formed in PAW and how they interact with cells without becoming toxic. Among the main agents generated in PAW is O_3_, a powerful oxidant that contributes to the formation of other RONS such as H_2_O_2_ and •OH, intensifying oxidative stress in exposed cells contributing to oxidative stress in exposed cells. These species can affect cellular components such as the cytoskeleton and adhesion properties, as observed in this study, possibly from complex physicochemical reactions involving the partial ionization of water molecules during plasma discharge, and their activity and stability are influenced by factors such as low pH, high oxidation-reduction potential and increased electrical conductivity. Under controlled conditions, RONS can interact with normal cells without causing damage, likely due to endogenous protective mechanisms (Estrella et al., 2013; Semmler et al., 2020). Tumor cells, which often present redox imbalance and reside in acidic microenvironments, are more susceptible to cumulative oxidative stress (Semmler et al., 2020; Xing et al., 2022; Xu et al., 2023; Jena et al., 2023). These discussions highlight the potential of PAW to induce differential responses depending on exposure time, RONS concentration, physicochemical control, and the biological nature of the target cells.

## 4 Conclusion

In this study, the PAW was generated using a dielectric barrier DBD system integrated with microbubble diffusion, specifically designed to enhance the transfer and solubilization of reactive species into the aqueous phase. The combination of plasma activation and microbubble bubbling resulted in efficient generation and distribution of RONS, including H_2_O_2_, O_3_, NO_2_^–^, NO_3_^–^, and HNO_2_, with concentrations increasing in a time-dependent manner. In particular, the accumulation of H_2_O_2_, O_3_, and HNO_2_ was accompanied by significant acidification of the solution, which likely contributed to the observed antimicrobial effects. The antimicrobial activity of PAW was systematically evaluated against three clinically relevant and resistant strains; *Staphylococcus aureus* (ATCC 6538), *Escherichia coli* (ATCC 25922), and *Candida albicans* (ATCC 10231), and the 14-min activated PAW demonstrated a pronounced bactericidal effect, achieving inhibition rates of ≥ 99% for *S. aureus* and *E. coli* and approximately 97% for *C. albicans*, while the 7-min PAW showed predominantly bacteriostatic responses. Additionally, cytotoxicity assessments on tumor (MCF-7, B16F10, J774) and non-tumor (L929, NOG-95) cell lines revealed selective effects, as PAW exposure caused significant morphological alterations and reduced viability in tumor cells, whereas non-tumor cells generally maintained viability above cytotoxic thresholds and preserved morphological integrity. Overall, these findings demonstrate that DBD-generated PAW with microbubble diffusion represents a promising antimicrobial strategy with potential applicability in biomedical fields such as medical device disinfection, wound treatment, and adjunctive therapies, warranting further studies aimed at optimizing activation parameters, improving long-term stability, and elucidating interactions with more complex biological systems.

## Data Availability

Requests to access the datasets should be directed to raquel.f.ferreira@unesp.br.
